# No relationship between the distribution of mast cells and the survival of stage IIIB colon cancer patients

**DOI:** 10.1186/1479-5876-9-88

**Published:** 2011-06-09

**Authors:** Qing Xia, Xiao-Jun Wu, Qiang Zhou, Jing-Zeng, Jing-Hui Hou, Zhi-Zhong Pan, Xiao-Shi Zhang

**Affiliations:** 1 State Key Laboratory of Oncology in South China, Sun Yat-sen University Cancer Center, Guangzhou 510060, China; 2 Biotherapy Center, Sun Yat-sen University Cancer Center, Guangzhou 510060, China; 3 Department of Colorectal Oncology, Sun Yat-sen University Cancer Center, Guangzhou 510060, China; 4 Department of Pathology, Sun Yat-sen University Cancer Center, Guangzhou 510060, China; 5 Department of Medical Oncology, The First People Hospital of Yueyang, Yueyang 414000, China

**Keywords:** Mast cells, Colon cancer, Survival, Progression

## Abstract

**Background:**

Mast cells promote the progression of experimental tumors and might be a valuable therapeutic target. However, the relevant clinical evidence is still controversial. This study analyzed the relationship between the distribution of mast cells and the survival of patients with colon cancer to study whether mast cells contribute to tumor progression.

**Materials and methods:**

Ninety-three cases of pathologically confirmed primary cancer tissues matched with adjacent normal mucosa, metastases of regional-draining lymph nodes and regional-draining lymph nodes without metastases were collected from stage IIIB colon carcinoma patients between January 1997 and July 2004 at the Cancer Center of Sun Yat-Sen University. Tryptase-positive mast cells were counted. The relationships of the distribution of mast cells with clinicopathologic parameters and 5-year survival were analyzed.

**Results:**

Although the mast cell count in the mucosa adjacent to the primary colon cancer was significantly higher than that in the stroma of the primary colon cancer, no difference in mast cell counts was observed between the stroma in lymph node metastasis and the lymph tissue adjacent to the metastasis. Additionally, the mast cell count in the regional-draining lymph node without the invasion of cancer cells was significantly higher than that in the stroma of lymph node metastasis and adjacent lymph tissue. However, none of those mast cell counts was related to 5-year survival.

**Conclusion:**

Although mast cell count varied with location, none of the mast cell counts was related to 5-year survival, suggesting that mast cells do not contribute to the progression of stage IIIB colon cancer.

## Background

In addition to the genetic alterations of cancer cells, it is believed that the infiltration of immune cells, such as dendritic cells, T cells, macrophages, and mast cells, are involved in the progression of colon cancer [1-6]. For example, mast cells might impact tumor progression by induction of angiogenesis, tissue remodeling, immune cell recruitment and direct cytotoxicity against cancer cells [7-9]. Because c-kit inhibitors such as imatinib and sunitinib have been approved in clinical practice and mast cells depend on c-kit, mast cells might be a new target for cancer therapy [10]. In animal models, polyps are infiltrated by pro-inflammatory mast cells and their precursors. Depletion of mast cells, either pharmacologically or through the generation of chimeric mice with genetic lesions in mast cell development, leads to a profound remission of existing polyps [11]. The interaction between mast cells and Treg cells shifts the local balance of immune surveillance in favor of tumor progression [12]. However, the relevant clinical evidence is controversial. For example, although Yodavudh and Nielsen reported that mast cell count was an independent prognostic factor for patients with colorectal cancer, this result was not confirmed by other groups [13-18].

Because these previous studies focused on the infiltration of mast cells into primary colorectal cancers and the function of mast cells might vary with their location in cancer tissue, it is reasonable to examine the distribution of mast cells and its relationship with the progression of colon cancer to identify the role of mast cells in this process. Therefore, the current study examined the mast cell counts in primary and metastatic tumors, as well as regional-draining lymph nodes without metastases, to study whether mast cells contribute to the progression of colon cancer.

## Materials and methods

### Materials

Ninety-three cases of pathologically confirmed primary tumor tissues matched with adjacent normal colon mucosa, metastases of regional-draining lymph nodes and regional-draining lymph nodes without metastases were collected from stage IIIB colon cancer patients between January 1997 and July 2004 at the Cancer Center of Sun Yat-Sen University. All the patients underwent radical surgery, and none of them had undergone either chemotherapy or radiotherapy before the collection of the samples. The histopathologic characteristics of the colon carcinoma tissue specimens were confirmed by blinded review of the original pathology slides. The TNM classification system of the American Joint Committee on Cancer (edition 7) was used for clinical staging, and the World Health Organization classification (2000 version) was used for pathologic grading. The study was conducted in accordance with the Helsinki Declaration and approved by the Ethics Committee of our institution. Patients were informed of the investigational nature of the study and provided their written informed consent.

### Follow-up of patients

Follow-up was provided to all of the patients. All patients were observed at 3-month intervals during the first year, once every 6 months in the second year, and by telephone or mail communication once every year thereafter for a total of 5 years. If recurrence or metastasis occurred, 5-Fu-based chemotherapy was administered according to the NCCN guidelines. Overall survival (OS) was defined as the time from surgery to death or was censored at the last known living date.

### Immunohistochemistry

The specimens were fixed in formaldehyde and embedded in paraffin. Tissue sections of 5 μm thickness were cut, dried, deparaffinized, and rehydrated in a series of alcohols and xylene before antigen retrieval by pressure cooker treatment in citrate buffer (pH 6.0) for 3 minutes. Then endogenous peroxidase was blocked with 3% hydrogen peroxide incubation. Mouse anti-human mast cell tryptase monoclonal antibody (at 1:160 000 dilution, Serotec, Oxford, UK) was used. Immunostaining was performed using an EnVision+ Dual Link Kit (DakoCytomation, Denmark) according to the manufacturer's instructions. The samples were developed with a substrate-chromogen solution [3,3'-diaminobenzidine dihydrochloride (DAB)] for 3-5 minutes. Sections were then counterstained with hematoxylin and mounted in non-aqueous mounting medium.

### Mast cell evaluation

The count of tryptase-positive mast cells in the cancer stroma of a primary tumor is denoted as MCC_stroma_. The stained sections were first screened under lower power (×100) to identify the areas with the most mast cells in the tumor stroma. MCC_stroma _was then counted under ×400 magnification (1 mm² per HP) in five fields of vision with an ocular micrometer. The number of mast cells in every field is expressed as MC/HP. Mean MCC_stroma _= total number of mast cells in the five fields divided by five. Additionally, the mast cell counts in the normal mucosa adjacent to the colon cancer (MCC_adjacent_), in the stroma of matched lymph node metastasis (MCC_slnm_), in the normal lymph tissue adjacent to the lymph node metastasis (MCC_alnm_) and in the regional-draining lymph node without metastasis (MCC_lnwm_) were evaluated as MCC_stroma_. All evaluated section were obtained from areas far from the area of necrosis and H.E. staining was reviewed in uncertain cases. The mast cell count in each section was scored independently by two pathologists with no prior knowledge of clinicopathologic parameters. The inter-observer agreement for the MCC was 81%. Disagreements were re-evaluated until a consensus was reached.

### Statistical analysis

Statistical analyses were performed using SPSS 13.0 software for Windows (SPSS Inc, Chicago, IL, USA). Descriptive statistical tests, including the mean, standard deviation, and median, were calculated according to standard methods. The relationships between the various clinicopathologic characteristics and the MCC parameters were compared and analyzed using chi-square tests, likelihood ratio, and linear-by-linear association, as appropriate. The non-parametric Wilcoxon signed ranks test and Kruskal-Wallis test were used to evaluate the significance of the differences of the mean ranks. Univariate and multivariate analyses were based on the Cox proportional hazards regression model. A two-tailed P < 0.05 was considered statistically significant.

## Results

### The distribution of mast cells

**Figure 1:**
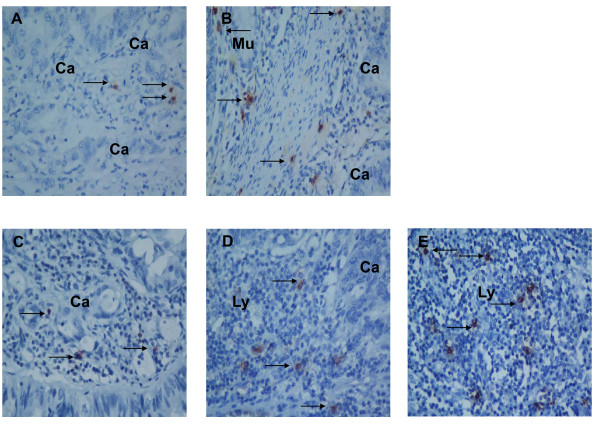
**The distribution patterns of mast cells in primary colon cancer, lymph node metastasis and normal regional-draining lymph node**. The tryptase-positive mast cells were stained using an immunohistochemical assay (×400). Higher frequencies of mast cells occurred in the mucosa adjacent to the colon cancer (MCC_adjacent_, Figure 1B) and in the regional-draining lymph node without metastasis (MCC_lnwm_, Figure 1E) than occurred in the lymph node metastasis (MCC_slnm _and MCC_alnm_, Figure 1C and Figure 1D) and the stroma of the primary colon cancer (MCC_stroma_, Figure 1A). Ca: cancer tissue; Ly: lymph node; Mu: colon mucosa.

The cytoplasm of mast cells stained brown. In primary tumor tissue, the mast cell count in normal mucosa adjacent to colon cancer (MCC_adjacent_) was significantly higher than that in the stroma of the primary colon cancer (MCC_stroma_) (P = 0.000). However, no difference in mast cell count was observed between the stroma in lymph node metastasis (MCC_slnm_) and the adjacent lymph tissue (MCC_alnm_) (P = 0.752). Additionally, the mast cell count in the regional-draining lymph node without metastasis (MCC_lnwm_) was significantly higher than that in the lymph tissue adjacent to lymph node metastasis (MCC_alnm_) (P = 0.000) (Figure 1 andTable 1).

**Table 1:** Mast cell counts in colon cancers

Location of mast cells	Mast cell count (median±interquartile range)
MCC_stroma*_	2.60 ± 4.80
MCC_adjacent_	10.60 ± 8.90
MCC_slnm_	4.00 ± 5.90
MCC_alnm_	5.20 ± 4.90
MCC_lnwm_	10.20 ± 10.00

*: MCC_stroma_, the count of tryptase-positive mast cells in the cancer stroma of the primary colon tumor; MCC_adjacent_, the count of tryptase-positive mast cells in the normal mucosa adjacent to the colon cancer; MCC_slnm_, the count of tryptase-positive mast cells in the stroma of matched lymph node metastasis; MCC_alnm_, the count of tryptase-positive mast cells in the normal lymph tissue adjacent to the lymph node metastasis; MCC_lnwm_, the count of tryptase-positive mast cells in the regional-draining lymph node without metastasis.

### Relationships between the distribution of mast cells and clinicopathologic characteristics

We used the chi-square test to assess the relationships between the distribution of mast cells and clinicopathologic characteristics. The results show that MCC_alnm _(the mast cell count in the normal lymph tissue adjacent to metastasis) was correlated with pathologic classifications and pathologic grades. MCC_alnm _was higher in papillary and tubular adenomas than that in mucoid and signet ring adenomas. Additionally, higher MCC_alnm _also occurred in lower-grade colon cancers, while higher MCC_lnwm _occurred in male patients (Table 2).

**Table 2:** Correlations between various MCCs and clinicopathologic characteristics

Variable	n	MCC_stroma* _	*P*	MCC_adjacent _	*P*	MCC_slnm_	*P*	MCC_alnm_	*P*	MCC_lnwm_	*P*
											
		<2.6 ≥2.6		<10.6≥10.6		<4.0 ≥4.0		<5.2 ≥5.2		<10.2 ≥10.2	
Age			0.243		0.911		0.377		0.121		0.471
< 60	43	18 25		21 22		22 21		25 18		23 20	
≥60	50	27 23		25 25		21 29		21 29		23 27	
Gender			0.407		0.574		0.726	26	0.250		**0.045 **
Male	58	30 28		30 28		26 32		26 32		24 34	
Female	35	15 20		16 19		17 18		20 15		22 13	
Location of primary tumor			0.431		0.336		0.094		0.588		0.472
Left	54	28 26		29 25		21 33		28 26		25 29	
Right	39	17 22		17 22		22 17		18 21		21 18	
Pathologic classification			0.732		0.652		0.900		**0.038 **		0.576
Papillary + tubular	73	36 37		37 36		34 39		32 41		35 38	
Mucoid + signet ring	20	9 11		9 11		9 11		14 6		11 9	
Pathologic Grade			0.799		0.998		0.991				0.582
G1	2	1 1		1 1		1 1		0 2	**0.001 **	1 1	
G2	69	32 37		34 35		32 37		28 41		32 37	
G3	22	12 10		11 11		10 12		18 4		13 9	
Growth type			1.000		0.769		0.239				0.769
Pushing	31	15 16		16 15		17 14		18 13		16 15	
Infiltrating	62	30 32		30 32		26 36		28 34	0.241	30 32	
Invasive depth			0.683		0.293		0.826				0.615
T3	77	38 39		40 37		36 41		39 38		39 38	
T4	16	7 9		6 10		7 9		7 9	0.615	7 9	

*: MCC_stroma_, the count of tryptase-positive mast cells in the cancer stroma of the primary colon tumor; MCC_adjacent_, the count of tryptase-positive mast cells in the normal mucosa adjacent to the colon cancer; MCC_slnm_, the count of tryptase-positive mast cells in the stroma of matched lymph node metastasis; MCC_alnm_, the count of tryptase-positive mast cells in the normal lymph tissue adjacent to the lymph node metastasis; MCC_lnwm_, the count of tryptase-positive mast cells in the regional-draining lymph node without metastasis.

### Survival analysis with univariate analysis

By the end of the 5-year follow-up, 66 patients with stage IIIB colon carcinoma were alive, so the 5-year survival rate was 70.9%. Based on univariate analysis, although the pathologic classification was a predictor of OS (P = 0.033), age, gender, location of primary tumor, pathologic grade, growth pattern, and tumor invasive depth showed no prognostic significance. More importantly, the mast cell counts in the primary tumor, metastasis and regional-draining lymph node without metastasis were not correlated with OS (Table 3).

**Table 3:** Univariate analysis of factors associated with OS

Variable	OS (n = 93)	
	
	HR, (95% CI)	*P*
Age (<60 y vs. ≥60 y)	0.635 (0.291-1.386)	0.249
Gender (female vs. male)	1.158 (0.537-2.495)	0.707
Location of primary tumor (right vs. left)	1.915 (0.896-4.093)	0.087
Pathologic classification (mucoid + signet ring vs. papillary + tubular)	2.325 (1.043-5.183)	**0.033 **
Pathologic grade (G3 vs. G2 + G1)	1.749 (0.785-3.894)	0.165
Growth type (infiltrating vs. pushing)	0.856 (0.392-1.870)	0.696
Invasive depth (T4 vs. T3)	0.853 (0.295-2.466)	0.768
MCC_stroma _*(≥2.6 MC/HP vs. <2.6 MC/HP)	1.224 (0.573-2.615)	0.600
MCC_adjacent _(≥10.6 MC/HP vs. < 10.6 MC/HP)	0.943 (0.443-2.006)	0.878
MCC_slnm _(≥4.0 MC/HP vs. < 4.0 MC/HP)	1.588 (0.727-3.469)	0.241
MCC_alnm _(≥5.2 MC/HP vs. <5.2 MC/HP)	1.045 (0.491-2.223)	0.909
MCC_lnwm _(≥10.2 MC/HP vs. <10.2 MC/HP)	0.779 (0.365-1.665)	0.518

*: MCC_stroma_, the count of tryptase-positive mast cells in the cancer stroma of the primary colon tumor; MCC_adjacent_, the count of tryptase-positive mast cells in the normal mucosa adjacent to the colon cancer; MCC_slnm_, the count of tryptase-positive mast cells in the stroma of matched lymph node metastasis; MCC_alnm_, the count of tryptase-positive mast cells in the normal lymph tissue adjacent to the lymph node metastasis; MCC_lnwm_, the count of tryptase-positive mast cells in the regional-draining lymph node without metastasis.

### Multivariate Cox proportional hazards analysis

Multivariate Cox proportional hazards analysis was used to determine whether the mast cell counts in the primary tumor, lymph node metastasis and normal regional-draining lymph node could serve as independent predictors of OS. Variables included age, gender, location of primary tumor, pathologic classification, pathologic grade, growth pattern, tumor invasive depth and the distributions of mast cells (MCC_stroma_, MCC_adjacent_, MCC_slnm_, MCC_alnm _and MCC_lnwm_). The results show that none of the variables was associated with OS (Table 4).

**Table 4:** Multivariate Cox analysis of factors associated with OS

Variable	OS (n = 93)	
	
	HR, (95% CI)	*P*
Age (<60 y vs. ≥60 y)	0.497 (0.219-1.127)	0.094
Gender (female vs. male)	1.302 (0.571-2.969)	0.531
Location of primary tumor (right vs. left)	2.220 (0.922-5.345)	0.075
Pathologic classification (mucoid + signet ring vs. papillary + tubular)	2.514 (0.662-9.537)	0.175
Pathologic grade (G3 vs. G2 + G1)	1.108 (0.300-4.094)	0.877
Growth type (infiltrating vs. pushing)	1.195 (0.489-2.917)	0.696
Invasive depth (T4 vs. T3)	1.456 (0.464-4.569)	0.520
MCC_stroma_*(≥2.6 MC/HP vs. <2.6 MC/HP)	1.180 (0.524-2.659)	0.690
MCC_adjacent _(≥10.6 MC/HP vs. < 10.6 MC/HP)	0.812 (0.372-1.774)	0.602
MCC_slnm _(≥4.0 MC/HP vs. < 4.0 MC/HP)	1.890 (0.748-4.773)	0.178
MCC_alnm _(≥5.2 MC/HP vs. <5.2 MC/HP)	0.916 (0.354-2.367)	0.856
MCC_lnwm _(≥10.2 MC/HP vs. <10.2 MC/HP)	0.729 (0.329-1.614)	0.436

*: MCC_stroma_, the count of tryptase-positive mast cells in the cancer stroma of the primary colon tumor; MCC_adjacent_, the count of tryptase-positive mast cells in the normal mucosa adjacent to the colon cancer; MCC_slnm_, the count of tryptase-positive mast cells in the stroma of matched lymph node metastasis; MCC_alnm_, the count of tryptase-positive mast cells in the normal lymph tissue adjacent to the lymph node metastasis; MCC_lnwm_, the count of tryptase-positive mast cells in the regional-draining lymph node without metastasis.

## Discussion

Multiple studies have analyzed the role of mast cells in the progression of primary colon cancer. Initial studies indicated that mast cell properties are independent prognostic factors [13,14]. However, this conclusion was questioned by subsequent studies [15-18]. Most of these studies have significant weaknesses, such as the mixture of colon with rectal cancers, the mixture of TNM stages, and small sample sizes [15-19]. This study analyzed 93 stage IIIB colon cancer patients to avoid those shortcomings. The results show that, although the mast cell count in the normal mucosa adjacent to the primary colon cancer (MCC_adjacent_) was higher than that in the stroma of the primary colon tumor (MCC_stroma_), neither MCC_adjacent _nor MCC_stroma _was correlated with the clinicopathologic parameters or 5-year survival rate. Therefore, in this patient population there was no direct evidence that infiltration of mast cells into primary cancer tissue impacted the progression of colon cancer. These results also refute the randomized distribution model of mast cells in cancer tissues suggested by Ribatti [20]. The reason that this kind of non-randomized distribution of mast cells would not impact the progression of colon cancer is unclear, it is possible that the role of mast cells was outweighed by that of angiogenesis, which is induced by multiple factors, including mast cells [21-23].

Since IIIB is a locally advanced stage and the potential effects of mast cells may be stronger in earlier stages of colon cancer development such as stage I, stage II and their function in metastatic disease may show quite different results. We analyzed this in the early research work and found the consistent result. Paraffin-embedded specimens, including tumor tissues and adjacent normal mucosa tissues obtained from 39 patients with pathologic evaluation-confirmed colon adenomas and 155 patients with colon cancers (the samples from stage I to IV were 38, 38, 38, 41), who underwent radical surgery or biopsy during the same period were analyzed using the same method. Results showed that the majority of mast cells were located in the normal mucosa adjacent to the colon cancer too, followed by the invasive margin and then cancer stroma. The mast cell count in the normal mucosa adjacent to the colon cancer was associated with the TNM classification characteristics and hepatic metastases, although it was not a prognostic factor. Otherwise, the mast cell count in the invasive margin was associated with neither the clinicopathlogic parameters nor overall survival, since the mast cell in the cancer stroma was rare, we didn't analyze it.

In addition to infiltrating primary tumors, mast cells also infiltrate metastases. The role of mast cells in metastasis is still not known. Therefore, this study examined the infiltration of mast cells in lymph node metastasis. In contrast to the infiltration of mast cells in the primary tumor, a similar distribution of mast cells occurred both in the stroma of lymph node metastasis (MCC_slnm_) and in the lymph tissue adjacent to the metastasis (MCC_alnm_). Although MCC_alnm _was higher in papillary and tubular adenomas than in mucoid and signet ring adenomas, and although higher MCC_alnm _occurred in lower-grade colon cancers, neither MCC_slnm _nor MCC_alnm _was correlated with 5-year survival, which suggests that mast cells are not involved in lymph node metastasis.

Because mast cells might impact tumor progression by regulating the immune microenvironment of regional-draining lymph nodes, this study also examined the mast cell count in the regional-draining lymph node without metastasis [24-27]. The results show that the mast cell count in this lymph node (MCC_lnwm_) was (10.20 ± 10.00)/HP, significantly higher than MCC_slnm _and MCC_alnm_. However, MCC_lnwm _was not correlated the 5-year survival, which again fails to support the hypothesis that mast cells contribute to the progression of colon cancer by an indirect mechanism.

Furthermore, the 5-year survival rate was 70.9% in our study, a little higher than an analysis of Surveillance, Epidemiology, and End Results (SEER) data (64.1%) [28]. Most of the cases were N1 status with 12 or more lymph nodes examined may help partially explain such a result. However, our study existed some limitations. For example, only 93 continual colon cancer patients were collected, sample was not big enough and there may be some selection bias thus further research is needed.

## Conclusion

By examining the distribution of mast cells in the primary tumor, in lymph node metastasis and in the normal regional-draining lymph node in 93 stage IIIB colon cancer patients, we found that, although the counts of mast cells varied with location, none of the mast cell counts was correlated with the 5-year survival rate. These data argue against the hypothesis that mast cells are involved in the progression of stage IIIB colon cancer.

## List of abbreviations used

MCC_adjacent _: the count of tryptase-positive mast cells in the normal mucosa adjacent to the colon cancer; MCC_alnm _: the count of tryptase-positive mast cells in the normal lymph tissue adjacent to the lymph node metastasis; MCC_lnwm _: the count of tryptase-positive mast cells in the regional-draining lymph node without metastasis; MCC_slnm _: the count of tryptase-positive mast cells in the stroma of matched lymph node metastasis; MCC_stroma _: the count of tryptase-positive mast cells in the cancer stroma of the primary colon tumor. OS: Overall survival;

## Competing interests

The authors declare that they have no competing interests.

## Authors' contributions

WXJ and PZZ performed the case collection. XQ and HJH performed the immunohistochemical staining. ZJ and ZQ analyzed the results. ZXS conceived the study, participated in the study design, and coordinated the writing and helped draft the manuscript. All authors read and approved the final manuscript.
